# Diagnostic value of contrast-enhanced ultrasound combined with other imaging techniques in differentiating benign and malignant thyroid nodules: a network meta-analysis

**DOI:** 10.3389/fendo.2026.1887134

**Published:** 2026-09-09

**Authors:** Qingying Xie, Zhuoshi Yang, Yue Sun, Na Zhao, Ruirui Hou, Zhiyue Di, Jian Ma

**Affiliations:** 1 Department of First Clinical Medical College, Heilongjiang University of Chinese Medicine, Harbin, Heilongjiang, China; 2 Endocrine Department I, The First Affiliated Hospital of Heilongjiang University of Chinese Medicine, Harbin, Heilongjiang, China

**Keywords:** benign and malignant, CEUS, imaging, network meta-analysis, thyroid nodules

## Abstract

**Background:**

This network meta-analysis (NMA) intends to fully assess the value of contrast-enhanced ultrasound (CEUS) combined with other imaging techniques in the diagnosis of thyroid nodules (TNs), and provide clinical guidance.

**Methods:**

We systematically searched PubMed, Web of Science, Embase, and Cochrane up to October 15, 2025, and assessed study quality by the QUADAS-2 tool. The combination of CEUS and CEUS with other techniques was assessed for accuracy by R4.5.1 and ANOVA-based NMA.

**Results:**

Twenty-six original studies were included, encompassing 5022 TNs from 4484 patients. CEUS-ultrasound elastography (UE) demonstrated favorable diagnostic performance in differentiating benign and malignant TNs (superiority index [SI]: 3.47, 95%CI 0.2–7). The pooled sensitivity (SE) and specificity (SP) of CEUS-UE were 0.92 (95%CI 0.88–0.95, SUCRA 80.0%) and 0.83 (95%CI 0.75–0.89, SUCRA 60.7%), respectively. CEUS-artificial intelligence (AI) was generally effective in identifying high-risk TNs (SI: 3.81, 95%CI 0.33–9), with pooled SE and SP of 0.95 (95%CI 0.91–0.98, SUCRA 90.0%) and 0.81 (95%CI 0.68–0.91, SUCRA 49.6%), respectively. CEUS-conventional ultrasound (US) showed favorable diagnostic performance in identifying non-risk-stratified TNs (SI: 5.29, 95%CI 0.33–9), with pooled SE and SP of 0.89 (95% CI 0.74–0.97, SUCRA 61.4%) and 0.88 (95%CI 0.69–0.97, SUCRA 76.9%), respectively.

**Conclusion:**

CEUS-UE demonstrates favorable overall diagnostic performance in differentiating benign and malignant TNs. CEUS-AI may offer additional value in identifying high-risk TNs with multiple suspicious features. CEUS-US is effective in identifying non-risk-stratified TNs. However, these subgroup findings require further validation in larger, diverse external cohorts.

**Systematic Review Registration:**

https://www.crd.york.ac.uk/PROSPERO/myprospero, identifier CRD420251169562.

## 1 Introduction

As medical imaging techniques advance, the detection rate of thyroid nodules (TNs) has reached 68% in clinical settings, displaying a rising trend recently (1). However, the overall malignancy rate of TNs remains as low as approximately 5%-15% (2). Due to the “high detection rate and low malignancy rate”, TN management has shifted from “detection of nodules” to “nodule risk stratification and precise treatment” (3). Early detection and early treatment are crucial for thyroid cancer prognosis, and research suggests that the five-year survival rate significantly rises following early treatment (4). Therefore, early and accurate differential diagnosis of benign and malignant TNs is significant for avoiding overtreatment, optimizing surgical decisions, and ameliorating patient outcomes.

Numerous studies have been conducted on CEUS combined strategies. CEUS has been increasingly applied to TN diagnosis. By intravenously injecting microbubble contrast agents, CEUS dynamically displays microvascular perfusion patterns within nodules and reflects their hemodynamic characteristics (5). It achieves differential diagnosis by qualitative and quantitative analyses. Qualitative analysis primarily involves fast-in, inhomogeneous enhancement, delayed enhancement, and hypoenhancement of malignant nodules during blood perfusion (5, 6). Regular annular enhancement is considered the most valuable indicator for diagnosing benign nodules (7, 8). Besides, quantitative analysis involves early washout time, a peak ratio <1.06, a sharpness ratio ≥1.60, a TTP ratio <1.15, blurred boundaries, irregular shape, and areas of hypoperfusion. A TTP ratio <1.15 may serve as an independent predictor of malignancy (8). However, the CEUS assessment protocol for TNs remains to be determined. Li et al. (9) found that regular annular hypoenhancement and hyperenhancement observed by CEUS can be found in both benign and malignant nodules. Zhang et al. (10) found that CEUS cannot identify PTC capsular extension in the case of equal enhancement due to changes in blood supply during tumor growth. Overall, the value of CEUS in distinguishing benign and malignant TNs remains limited. Additionally, CEUS serves more as an adjunct to standard ultrasound for risk stratification than as a substitute in many clinical settings. Therefore, CEUS is suitable as a basis for imaging assessment, but it is also necessary to assess the incremental value of CEUS combined with other imaging techniques.

A retrospective study revealed that when distinguishing C-TIRADS grade 4–5 nodules from PTC, the AUC of CEUS and dynamic AI was 0.86, while the AUC of the combination of the two was 0.83 (11). Other studies showed that CEUS-SWE can improve diagnostic performance, with an AUC of 0.84 for CEUS alone and 0.9 for CEUS-SWE (6). However, diagnostic values of CEUS alone and its combined strategies are inconsistent across studies, with a lack of head-to-head comparisons of techniques. Network meta-analysis (NMA), an advanced statistical method, enables simultaneous comparison and ranking of three or more diagnostic strategies, thereby offering more comprehensive and reliable evidence-based guidance.

Therefore, this study intends to fully assess the value of CEUS and CEUS combined with different diagnostic techniques (including CEUS-TIRADS, CEUS-UE, CEUS-AI, and CEUS-US) in the differential diagnosis of benign and malignant TNs by an NMA of diagnostic trials. We do not seek to claim a universally optimal strategy, but to provide a combined imaging strategy based on CEUS that is closer to clinical practice.

## 2 Methods

This study followed the PRISMA guidance (12) and was registered with PROSPERO (CRD420251169562). All included studies received ethical approval in the original articles.

### 2.1 Search strategy

We systematically searched PubMed, Web of Science, Embase, and Cochrane up to October 15, 2025, with no restrictions on the region, publication status, and language. Medical subject headings plus free-text terms were utilized, such as “thyroid nodule”, “thyroid gland nodule”, “colloid nodule (thyroid)”, “nodule of the thyroid gland”, “contrast-enhanced ultrasound”, “CEUS”, and “contrast-enhanced ultrasonography”. The search formula is detailed in **Supplementary Table 1**. The references of relevant studies were also checked to prevent omission.

### 2.2 Eligibility criteria

Inclusion criteria: (1) population: suspected patients of malignant TNs, no age restrictions; (2) diagnostic tools: CEUS alone and CEUS-TIRADS, UE, AI, or US; (3) gold standard: pathology; (4) outcome measures: “2×2 tables” of true positive (TP), false positive (FP), false negative (FN), and true negative (TN), or relevant metrics for calculation; and (5) study design: No restrictions.

Exclusion criteria: (1) reviews, conference abstracts, meta-analyses, letters, case reports, guidelines, and consensus; (2) animal studies; (3) unavailable full texts or data; (4) qualitative studies lacking core data; and (5) machine learning models.

### 2.3 Study screening

Two investigators (X.Q.Y. and S.Y.) independently screened studies based on the eligibility criteria. Disagreements were resolved by discussion with a third investigator. First, all retrieved studies were imported into EndNote21. After duplicate publications were removed, titles and abstracts were screened. The potentially eligible studies were then examined for full text to finally include the eligible studies.

### 2.4 Data extraction

Two investigators (X.Q.Y. and S.Y.) collected basic information of the included studies: first author, publication year, study type, country, population, patient age, sex, gold standard, diagnostic technique, and equipment used. Diagnostic techniques primarily included CEUS, CEUS-TIRADS, CEUS-UE, CEUS-AI, and CEUS-US.

Then TP, FP, FN, and TN were extracted from each study. If the study did not directly provide these data but reported SE, SP, and the number of patients and TNs, the standard 2×2 contingency table could be simply calculated. It should be noted that the number of patients may not match that of TNs. During data processing, the names of diagnostic techniques were converted to corresponding numerical codes.

Two investigators (X.Q.Y. and S.Y.) independently conducted data extraction, and disagreements were resolved by discussion with a third investigator.

### 2.5 Quality assessment

Two investigators (X.Q.Y. and S.Y.) assessed study quality and applicability using the QUADAS-2 tool (13) from four domains for risk of bias (RoB) (patient selection, index test, reference standard, and flow and timing) and three domains for applicability (patient selection, index test, and reference standard). The RoB was assessed as low if all answers to the questions within a domain were “Yes”, or as high if all answers within any domain were “No”. The RoB was judged as unclear in the case of insufficient information. Disagreements were resolved by discussion or consultation with a third investigator (Z.N.). RevMan5.4 (The Cochrane Collaboration) was utilized to record the study quality and generate plots.

### 2.6 Data analysis

Bayesian NMA of diagnostic test accuracy (NMA-DTA) was performed using R software (v4.5.1, R Foundation for Statistical Computing, Vienna, Austria). We adopted the ANOVA model developed by Nyaga et al. (14) as the primary model. This model was used to integrate direct and indirect evidence across diverse diagnostic techniques and assess the relative diagnostic performance of CEUS alone versus CEUS combined with different diagnostic techniques for differentiating benign and malignant TNs. To unify heterogeneous diagnostic thresholds across the included studies, sensitivity and specificity of studies were logit-transformed. On this basis, sensitivity and specificity were jointly modeled, and study-level random effects were incorporated to account for the inherent negative correlation between these two indicators, effectively minimizing threshold-induced bias.

Bayesian estimation was implemented via the Rstan package using Markov Chain Monte Carlo (MCMC) sampling with two chains (iter=10,000; warmup=5000; thin=5). Default non-informative priors were applied to avoid interference with parameter estimation. Model convergence was evaluated using the R-hat statistic, with R-hat <1.1 defined as good convergence. Trace plots were visually inspected to further confirm model stability (**Supplementary Figure 1**). The model assumed consistent effect parameters for diagnostic odds ratio (DOR) across comparisons. DOR-based ranking was used to reflect the discriminatory power of each test. Higher DOR values indicated higher discriminatory power. The primary outcome measures included pooled sensitivity, specificity, and AUC, with 95% CIs.

The AUC was calculated using the hierarchical summary receiver operating characteristic model. The model was generally logit-transformed. When AUC approaches 1.0 (e.g., >0.95), its corresponding 95% CI may exceed 1.0.

We further introduced the superiority index proposed by Deutsch et al. (15) to quantify the relative diagnostic performance of each test by synthesizing sensitivity and specificity. The superiority index is defined as follows:


$$Superiority\;index_{k}=\frac{2a_{k}+c_{k}}{2b_{k}+c_{k}}$$

where *a_k_* = number of tests superior to the test *k* in both sensitivity and specificity;

*b_k_* = number of tests inferior to the test *k* in both sensitivity and specificity;

*c_k_* = number of tests with identical sensitivity and specificity to the test *k*.

A network diagram was generated using the R gemtc package. Consistency and inconsistency models were developed to evaluate the coherence of network evidence. For networks with closed loops, the node-splitting method was applied to detect local inconsistency between direct and indirect evidence (**Supplementary Figures 2**, **S3**). For networks without closed loops, the local inconsistency test was not feasible due to the absence of direct and indirect evidence available for comparisons. Accordingly, model fit and deviance information criterion (DIC) values were compared between consistency and inconsistency models for auxiliary assessment.

The surface under the cumulative ranking curve (SUCRA, 0%-100%) was calculated to rank the sensitivity and specificity of each diagnostic technique, with higher values representing higher rankings. SUCRA values were calculated independently within each network and subgroup, and were not used to directly compare diagnostic techniques across networks or subgroups. A fixed-effects model was applied for low heterogeneity (I²=25%) and a random-effects model for moderate-to-high heterogeneity (I²=50% and 75%). Fuzzy priors were utilized for analysis using gemtc, and model convergence was evaluated using the potential scale reduction factor (PSRF), with PSRF<1.05 indicating good convergence (**Supplementary Table 2**).

### 2.7 Subgroup analysis

The patients were grouped into high-risk and non-risk-stratified populations to explore heterogeneity. The former was defined as TIRADS grade 4-5, Bethesda V-VI, suspicious signs, and scheduled surgery. The latter was defined as TIRADS grade 1–5 and absence of special description. Other potential sources of heterogeneity, such as machine model, study type, and country, were also examined.

## 3 Results

### 3.1 Search results and flowchart

**Figure 1:**
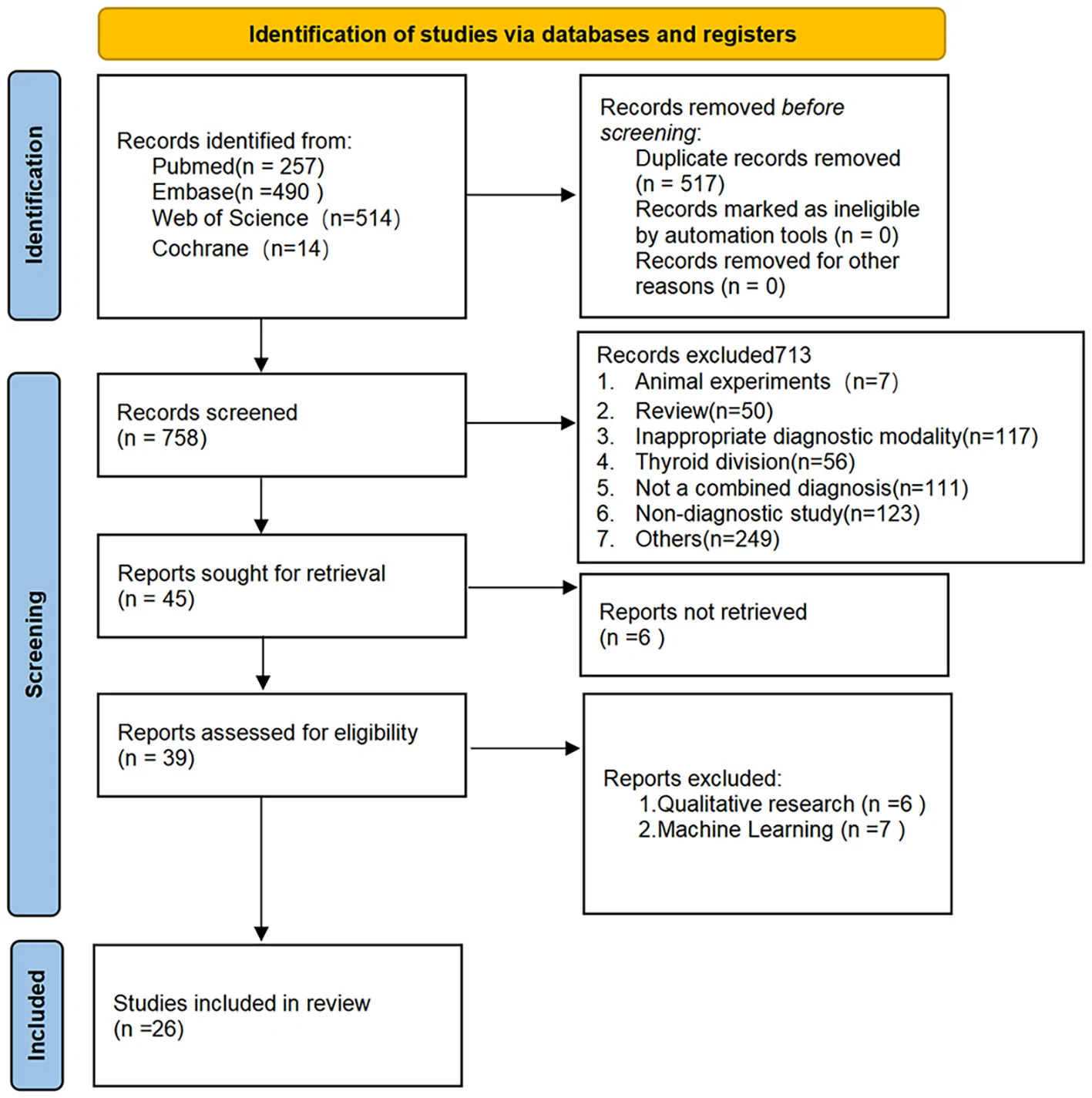
Screening flowchart.

We initially retrieved 1,275 studies from PubMed, Web of Science, Embase, and Cochrane. After duplicates were removed, 758 studies were obtained. Another 713 studies were excluded after initial screening based on the eligibility criteria. The full text was examined for the remainder, and seven studies were excluded due to the use of machine learning models, and six due to being qualitative studies lacking core data. Ultimately, 26 studies (6, 7, 11, 16–38) were included (**Figure 1**).

### 3.2 Basic characteristics

The included studies encompassed 5022 TNs from 4484 patients, including 2370 benign nodules and 2652 malignant nodules. 24 studies were from China, one from India, and one from Romania. There were 7 prospective cohort studies and 19 retrospective cohort studies. Except for two studies lacking information of sex, the remaining studies involved 1140 men and 3198 women. Patients were aged 15–87 years, with an average of approximately 46.5 years, but 10 studies lacked information of age. Five diagnostic techniques were adopted: CEUS, CEUS-TIRADS, CEUS-UE, CEUS-AI, and CEUS-US. Commonly used machines included Philips, GE LOGIQ, Siemens, Esaote, and SuperSonic (**Table 1**).

**Table 1:** Study characteristics.

Author	Year	Country	Study type	Population type	Male/female	Age	Number of participants	Number of TNs	Machine
Zhang (6)	2025	China	Retrospective cohort	CTIRADS:4	66/153	45.0 ± 13.20	219	219	CEUS: Esaote /SWE: Supersonic
You (11)	2025	China	Retrospective cohort	CTIRADS:4-5	117/221	/	338	378	CEUS: Hitachi/AI: GE LOGIQ
Yan (16)	2025	China	Retrospective cohort	Bethesda: V-VI	39/113	48.10 ± 13.10	152	166	GE LOGIQ; Philips IU-22
Liu (18)	2024	China	Retrospective cohort	Bethesda: III-V	41/75	51.96 ± 13.56	116	116	Toshiba Aplio 500
Zou (19)	2024	China	Retrospective cohort	Unassorted	25/184	45	209	268	Samsung RS85/Mindray
Zhang (20)	2024	China	Retrospective cohort	Bethesda: III-V	40/152	47 ± 13.50	192	192	Esaote
Verma (21)	2023	India	Prospective cohort	Unassorted	25/25	/	50	52	Philips IU-22
Gong (22)	2022	China	Retrospective cohort	ACRTIRADS:4	35/113	49.94 ± 15.34	140	148	CEUS: Siemens/AI: GE LOGIQ
Andreea (23)	2024	Romania	Prospective cohort	EUTIRADS:3–5	13/120	48	133	157	SuperSonic
Zhu (24)	2022	China	Prospective cohort	CTIRADS:4	/	/	100	100	Philips IU22
Wang (25)	2022	China	Retrospective cohort	Unassorted	105/448	/	553	600	Philips iU22 /Siemens
Wang (26)	2023	China	Retrospective cohort	ACRTIRADS:4-5	19/114	/	113	133	Supersonic
Jin (27)	2022	China	Retrospective cohort	CTIRADS:3-5	37/76	47.20 ± 12.20	113	116	GE LOGIQ/Samsung RS80A
Xu (28)	2020	China	Retrospective cohort	Scheduled for surgery	28/54	/	82	96	Siemens/Supersonic
Zhao (29)	2019	China	Retrospective cohort	Suspicious signs	30/87	/	108	117	GE LOGIQ
Xu (30)	2018	China	Retrospective cohort	ACRTIRADS:3-4	68/302	43.20 ± 11.70	370	432	Philips IU22
Lin (31)	2019	China	Retrospective cohort	No diagnosis by FNA	87/139	/	226	236	Esaote
Zhao (32)	2019	China	Retrospective cohort	Unassorted	32/111	45.40 ± 10.80	143	150	GE LOGIQ
He (33)	2018	China	Prospective cohort	Maximal diameter ≥6 mm	/	46 ± 15.20	83	88	GE LOGIQ/Siemens
Zhang (34)	2016	China	Retrospective cohort	Unassorted	85/161	46.10 ± 15.20	246	319	Siemens
Li (17)	2017	China	Retrospective cohort	Calcified	21/68	43.20 ± 1.80	89	89	Philips iU22
Zhang (7)	2016	China	Prospective cohort	Scheduled for surgery	20/91	/	111	145	Esaote
Sui (35)	2016	China	Prospective cohort	Scheduled for surgery	50/47	48.60 ± 12.40	97	109	CEUS: Philips IU22/RTE: Siemens
Liu (36)	2016	China	Retrospective cohort	RTIRADS: 3-4	29/73	/	102	102	Esaote
Chen (37)	2016	China	Retrospective cohort	Scheduled for surgery	86/167	43 ± 12	253	319	SWE: SuperSonic/CEUS: Philips iU22
Deng (38)	2014	China	Prospective cohort	Unassorted	42/104	46.3 ± 12.5	146	175	Siemens

RTIRADS: TI-RADS proposed by Russ et al. (51).

### 3.3 Quality assessment

**Figure 2:**
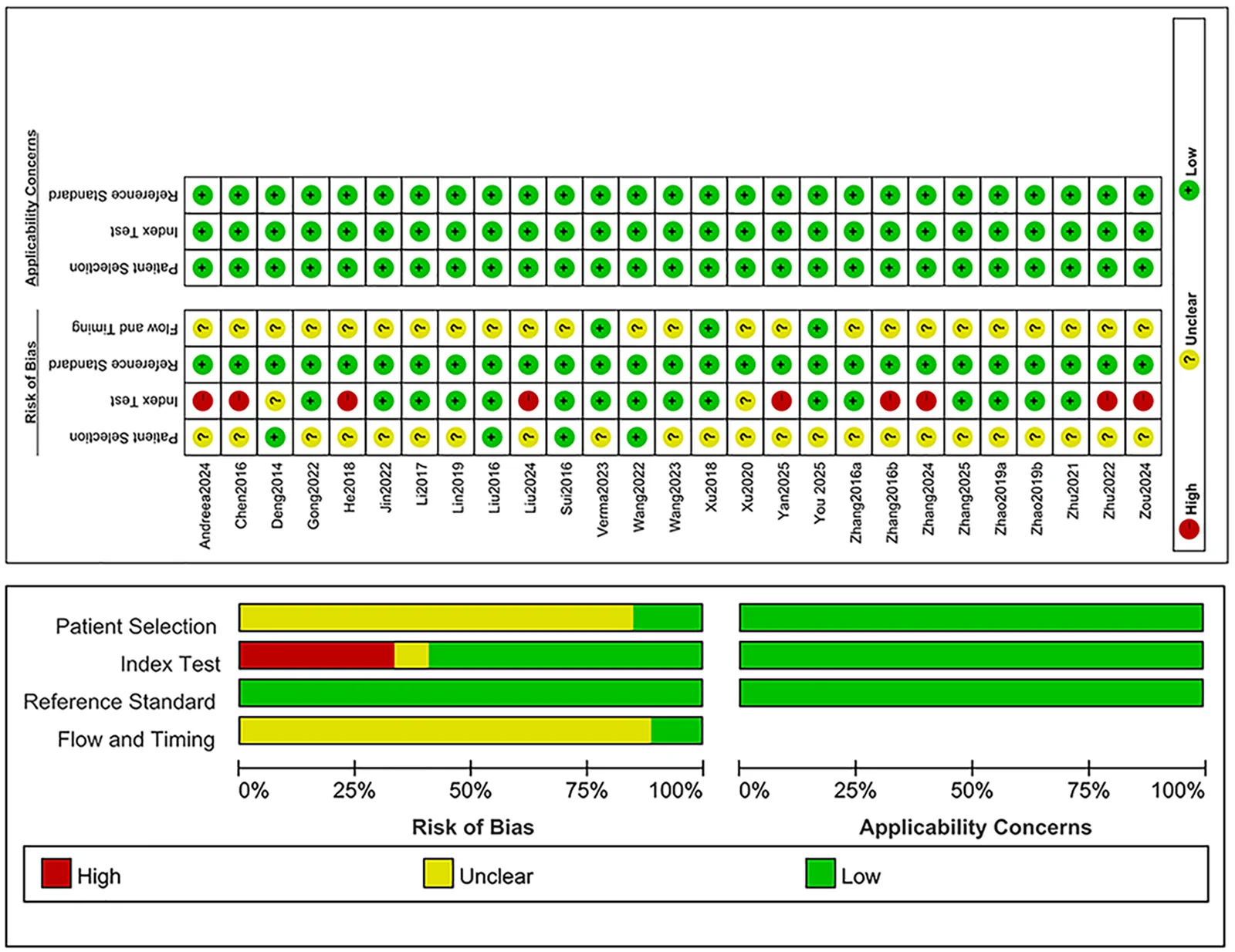
Dot plot for quality assessment results (upper); bar chart for publication bias (lower) (left: RoB; right: applicability).

Using the QUADAS-2 tool, 17 high-quality studies and 9 medium-quality studies were identified, demonstrating strong clinical applicability (**Figure 2**). 22 studies did not explicitly report whether patients were enrolled randomly or consecutively, resulting in unclear RoB in the patient selection. Nine studies did not predefine thresholds, producing high RoB in the index test. Two studies did not clearly state the risk of bias, so the risk of bias was unclear. 23 studies provided no explicit details, leading to unclear RoB in the flow and timing.

### 3.4 Performance of different methods in differential diagnosis of benign and malignant TNs

All included studies set up a control group of CEUS alone. CEUS-TIRADS, CEUS-UE, CEUS-AI, and CEUS-US were adopted in twelve, eight, five, and four studies, respectively. Specifically, CEUS alone yielded a pooled SE of 0.83 (95%CI 0.79-0.86), and a pooled SP of 0.81 (95%CI 0.77-0.85), with an AUC of 0.91 (95%CI 0.89-0.93). All combined strategies were superior to CEUS alone in differential diagnosis: CEUS-TIRADS with a pooled SE of 0.92 (95%CI 0.89-0.94), a pooled SP of 0.80 (95%CI 0.74-0.86), and an AUC of 0.95 (95%CI 0.91-1.00); CEUS-UE with a pooled SE of 0.92 (95%CI 0.88-0.95), a pooled SP of 0.83 (95%CI 0.75-0.89), and an AUC of 0.97 (95%CI 0.96-0.99); CEUS-AI with a pooled SE of 0.93 (95%CI 0.87-0.96), a pooled SP of 0.80 (95%CI 0.69-0.88), and an AUC of 0.95 (95%CI 0.78-1.23); CEUS-US with a pooled SE of 0.88 (95%CI 0.80-0.93), a pooled SP of 0.90 (95%CI 0.83-0.95), and an AUC of 0.96 (95%CI 0.94-0.99) (**Table 2**).

**Table 2:** Performance of different methods in differential diagnosis of benign and malignant TNs.

Diagnostic techniques	SE (95%CI)	SP (95%CI)	DOR (95%CI)	SI (95%CI)	Relative SE (95%CI)	Relative SP (95%CI)
CEUS	0.83 (0.79-0.86)	0.81 (0.77-0.85)	21.73 (15.19-30.37)	0.21 (0.11-0.33)	1	1
CEUS-TIRADS	0.92 (0.89-0.94)	0.80 (0.74-0.86)	46.99 (26.96-77.66)	1.30 (0.14-5)	1.11 (1.06-1.16)	0.99 (0.91-1.05)
CEUS-UE	0.92 (0.88-0.95)	0.83 (0.75-0.89)	64.75 (30.71-122.24)	3.47 (0.20-7)	1.11 (1.06-1.17)	1.02 (0.93-1.10)
CEUS-AI	0.93 (0.87-0.96)	0.80 (0.69-0.88)	60.92 (23.45-135.56)	2.48 (0.14-7)	1.12 (1.05-1.18)	0.98 (0.86-1.09)
CEUS-US	0.88 (0.80-0.93)	0.90 (0.83-0.95)	78.89 (28.36-172.83)	3.37 (1.00-9)	1.06 (0.97-1.14)	1.11 (1.02-1.18)

**Figure 3:**
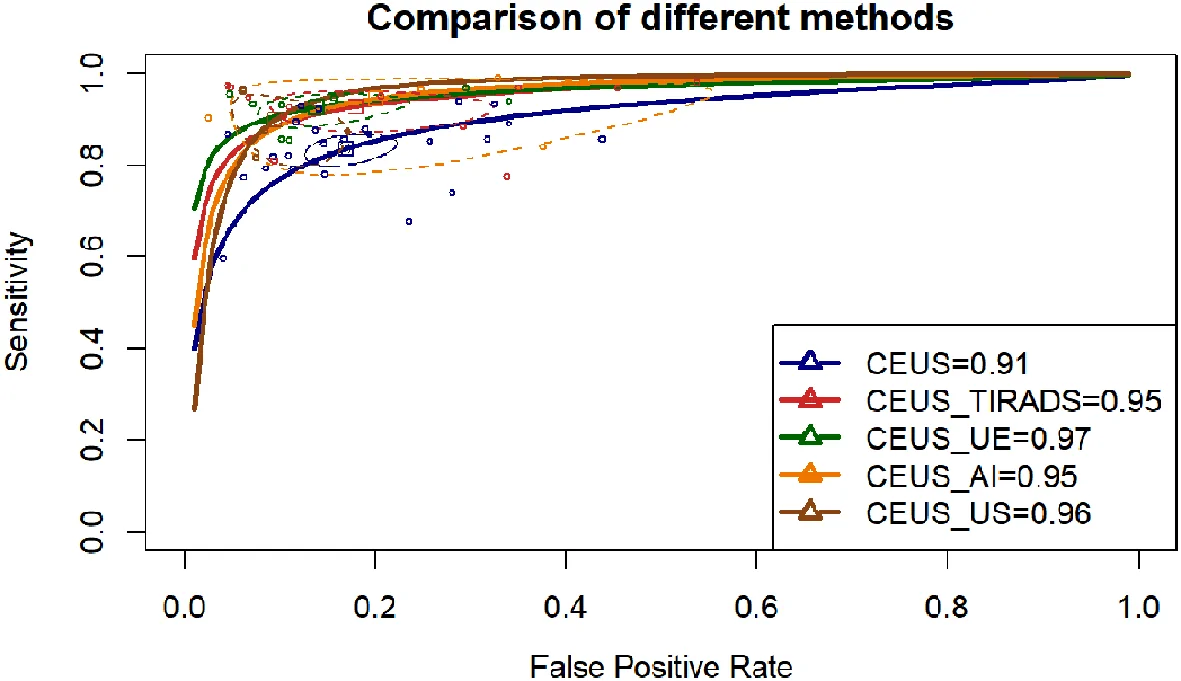
AUC values of CEUS and CEUS combined strategies in differentiating benign and malignant TNs. The steepness of the ROC curve reflects the discriminatory power of the model at different thresholds, especially its capability to balance the TP and FP.

CEUS-UE had the highest SI (3.47, 95%CI 0.20-7), followed by CEUS-US (3.37, 95%CI 1-9), CEUS-AI (2.48, 95%CI 0.14-7), CEUS-TIRADS (1.30, 95%CI 0.14-5), and CEUS (0.21, 95%CI 0.11-0.33). CEUS-US had the highest DOR (78.89, 95%CI 28.36-172.83), followed by CEUS-UE (64.75, 95%CI 30.71-122.24), CEUS-AI (60.92, 95%CI 23.45-135.56), CEUS-TIRADS (46.99, 95%CI 26.96-77.66), and CEUS (21.73, 95%CI 15.19-30.37) (**Figure 3**).

**Figure 4:**
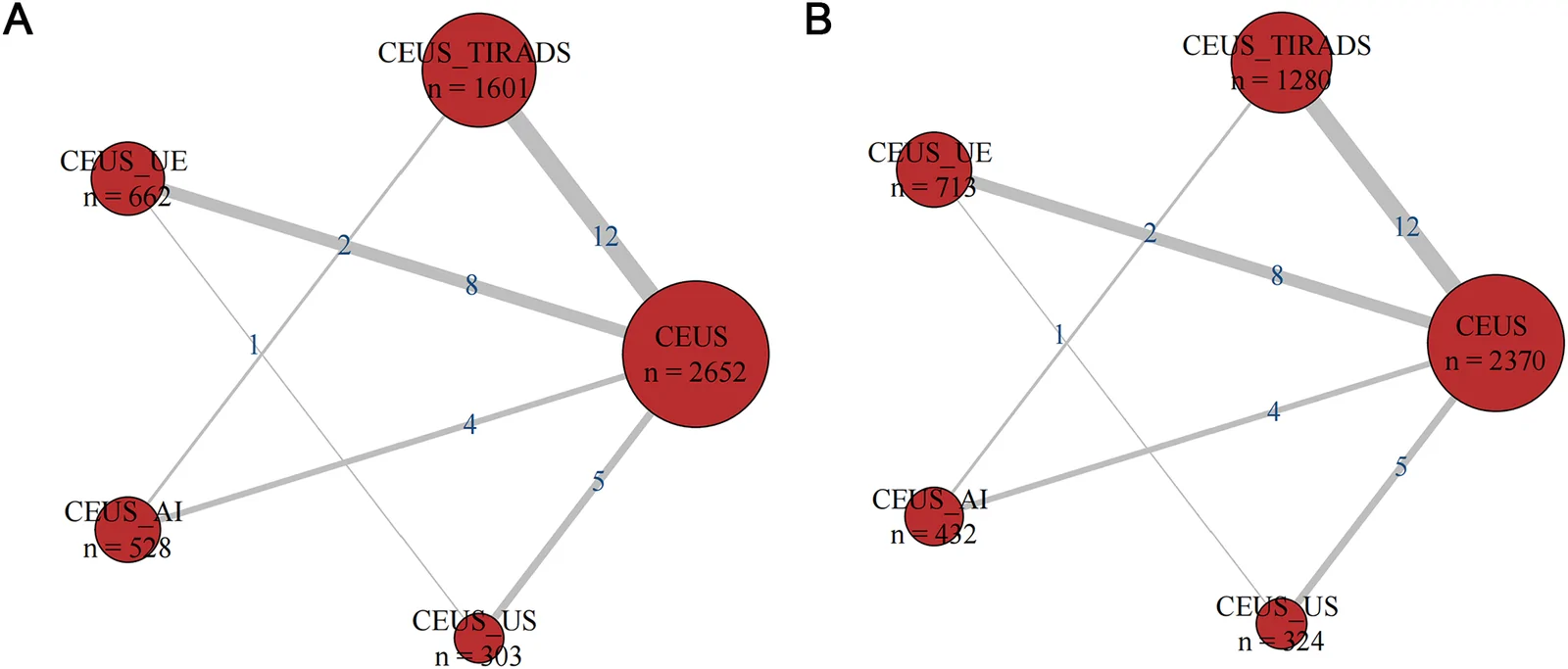
**(A)** Network diagram for SE in differentiating benign and malignant TNs; **(B)** Network diagram for SP in differentiating benign and malignant TNs.

SUCRA ranking and pairwise comparisons using league tables were performed for SE and SP, and evidence networks were generated (**Figure 4**). For diagnostic SE, CEUS-UE and CEUS-AI achieved the highest SUCRA value (80%), followed by CEUS-TIRADS (60.8%), CEUS-US (21.6%) and CEUS alone (7.6%). As shown in **Supplementary Table 3**, CEUS-AI had slightly higher SE than CEUS-UE, indicating its slight advantage in identifying malignant TNs.

For diagnostic SP, CEUS-US had the highest SUCRA value (99.9%), followed by CEUS-UE (60.7%), CEUS (51%), CEUS-AI (21.8%), and CEUS-TIRADS (16.5%). The corresponding league table are presented in **Supplementary Table 4**.

Moderate between-study heterogeneity was detected (I²=61% for SE and I²=60.3% for SP). The inconsistency test demonstrated no significant inconsistency between direct and indirect evidence across the network (**Supplementary Figure 2**).

To sum up, the CEUS combined strategies enhanced diagnostic efficacy. CEUS-AI demonstrated the highest SE, exhibiting strong capability in identifying malignant nodules, while CEUS-US had the highest SP, demonstrating the strongest ability to exclude interference of benign nodules. CEUS-UE was significantly effective in distinguishing benign and malignant TNs, particularly in the complementary indicator system.

### 3.5 Subgroup analysis

#### 3.5.1 Performance of different methods in identifying high-risk TNs

**Figure 5:**
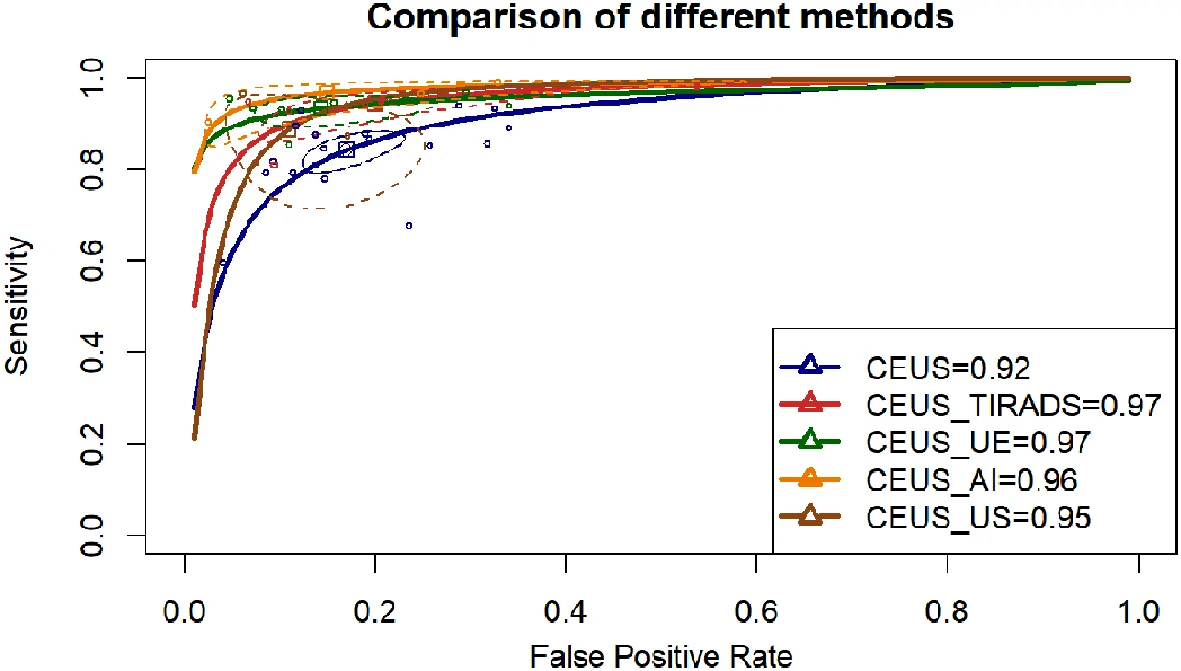
AUC values of CEUS and CEUS combined strategies in differentiating suspicious high-risk TNs.

The high-risk population was included in 18 studies. Overall, SE and SP varied across combined strategies. The pooled SE was highest for CEUS-AI (0.95, 95%CI 0.91-0.98), followed by CEUS-UE (0.94, 95%CI 0.90-0.96) and CEUS-TIRADS (0.92, 95%CI 0.88-0.96). The pooled SE was lower for CEUS (0.83, 95%CI 0.79-0.87) and CEUS-US (0.84, 95%CI 0.72-0.92). Besides, CEUS-US had the highest pooled SP (0.90, 95%CI 0.80-0.96), followed by CEUS (0.81, 95%CI 0.75-0.86), CEUS-UE (0.81, 95%CI 0.71-0.88), CEUS-AI (0.81, 95%CI 0.68-0.91), and CEUS-TIRADS (0.78, 95%CI 0.68-0.86). The AUC also varied among strategies: 0.96 (95%CI 0.97-1.00) for CEUS-AI, 0.97 (95%CI 0.96-0.99) for CEUS-UE, 0.97 (95%CI 0.95-0.99) for CEUS-TIRADS, 0.92 (95%CI 0.91-0.93) for CEUS, and 0.95 (95%CI 0.73-1.36) for CEUS-US (**Figure 5**).

CEUS-AI had the highest SI (3.81, 95%CI 0.33-9), followed by CEUS-UE (2.70, 95%CI 0.20-7), CEUS-US (2.03, 95%CI 0.20-5), CEUS-TIRADS (1.06, 95%CI 0.20-5), and CEUS (0.31, 95%CI 0.11-1). Similarly, the DOR was the highest for CEUS-AI (109.05, 95%CI 33.80-272.61), followed by CEUS-UE (72.13, 95%CI 31.08-134.04), CEUS-US (61.74, 95%CI 17.87-161.82), CEUS-TIRADS (49.54, 95%CI 21.55-94.59), and CEUS (22.09, 95%CI 14.58-32.05) (**Table 3**).

**Table 3:** Performance of different methods in identifying high-risk TNs.

Diagnostic techniques	SE (95%CI)	SP (95%CI)	DOR (95%CI)	SI (95%CI)	Relative SE (95%CI)	Relative SP (95%CI)
CEUS	0.83 (0.79-0.87)	0.81 (0.75-0.86)	22.09 (14.58-32.05)	0.31 (0.11-1)	1	1
CEUS-TIRADS	0.92 (0.88-0.96)	0.78 (0.68-0.86)	49.54 (21.55-94.59)	1.06 (0.20-5)	1.11 (1.05-1.17)	0.97 (0.85-1.08)
CEUS-UE	0.94 (0.90-0.96)	0.81 (0.71-0.88)	72.13 (31.08-134.04)	2.70 (0.20-7)	1.13 (1.07-1.19)	1.00 (0.89-1.10)
CEUS-AI	0.95 (0.91-0.98)	0.81 (0.68-0.91)	109.05 (33.80-272.61)	3.81 (0.33-9)	1.15 (1.09-1.21)	1.00 (0.84-1.13)
CEUS-US	0.84 (0.72-0.92)	0.90 (0.80-0.96)	61.74 (17.87-161.82)	2.03 (0.20-5)	1.01 (0.88-1.12)	1.11 (0.98-1.22)

**Figure 6:**
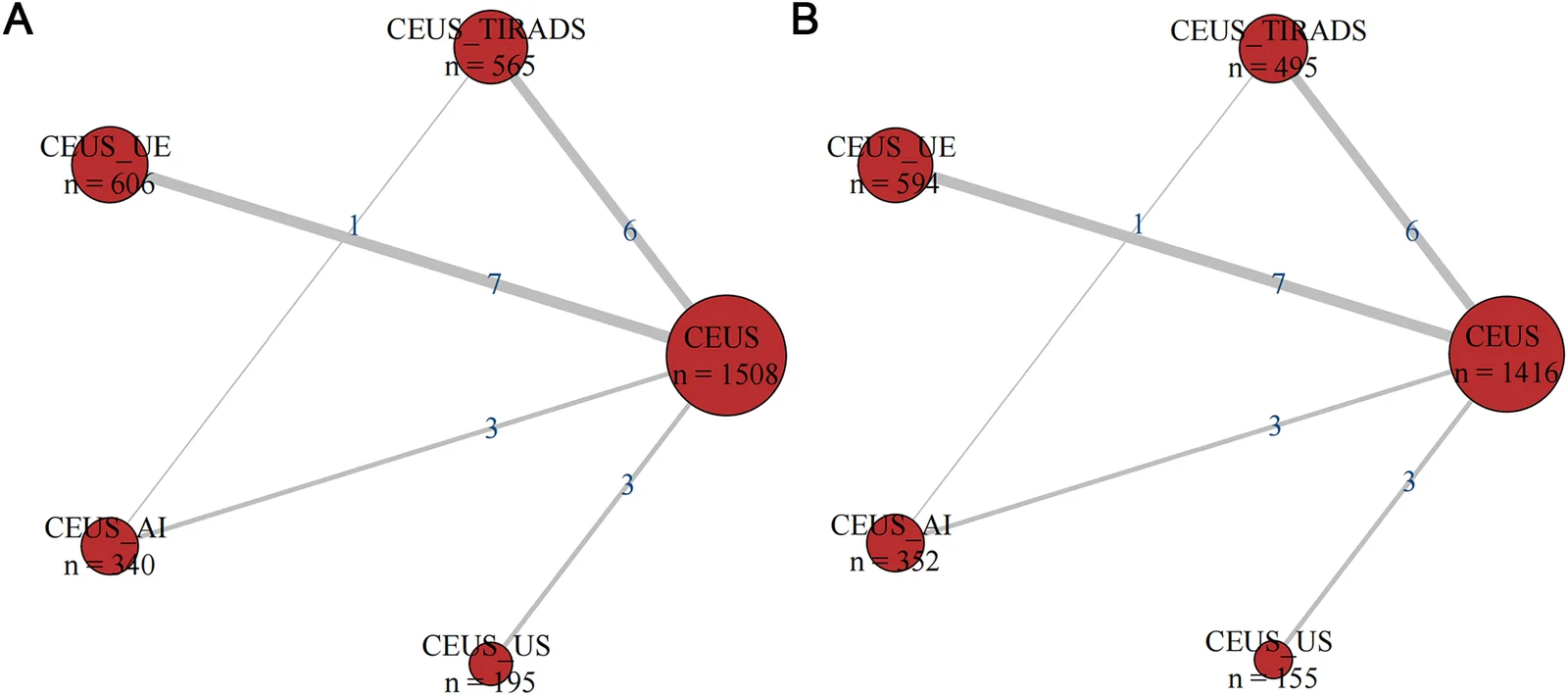
**(A)** Network diagram for SE in identifying high-risk TNs; **(B)** Network diagram for SP in identifying high-risk TNs.

SUCRA values were calculated for SE and SP. For SE, CEUS-AI had the highest SUCRA value (90%), followed by CEUS-UE (82.7%), CEUS-TIRADS (52.3%), CEUS (21%), and CEUS-US alone (4.1%). These results suggested that CEUS-AI might rank first for SE in identifying high-risk TNs. The corresponding network diagram and league table are presented in **Figure 6A** and **Supplementary Table 5**, respectively.

For SP, CEUS-US achieved the highest SUCRA value (95.1%), followed by CEUS-AI (49.6%), CEUS alone (41.3%), CEUS-UE (34.7%), and CEUS-TIRADS (29.4%). The corresponding network diagram and league table are shown in **Figure 6B** and **Supplementary Table 6**, respectively.

We found low-to-moderate between-study heterogeneity for SE (I²=36.5%) and substantial heterogeneity for SP (I²=68.8%). No significant inconsistency was detected between direct and indirect evidence (**Supplementary Figure 3**).

Considering SE, SP, AUC, SI, SUCRA, and DOR, CEUS combined strategies generally possessed a stronger capability in identifying high-risk TNs. Different combined strategies exhibited distinct advantages, with CEUS-AI being superior in SE and overall discrimination.

#### 3.5.2 Performance of different methods in identifying non-risk-stratified TNs

**Figure 7:**
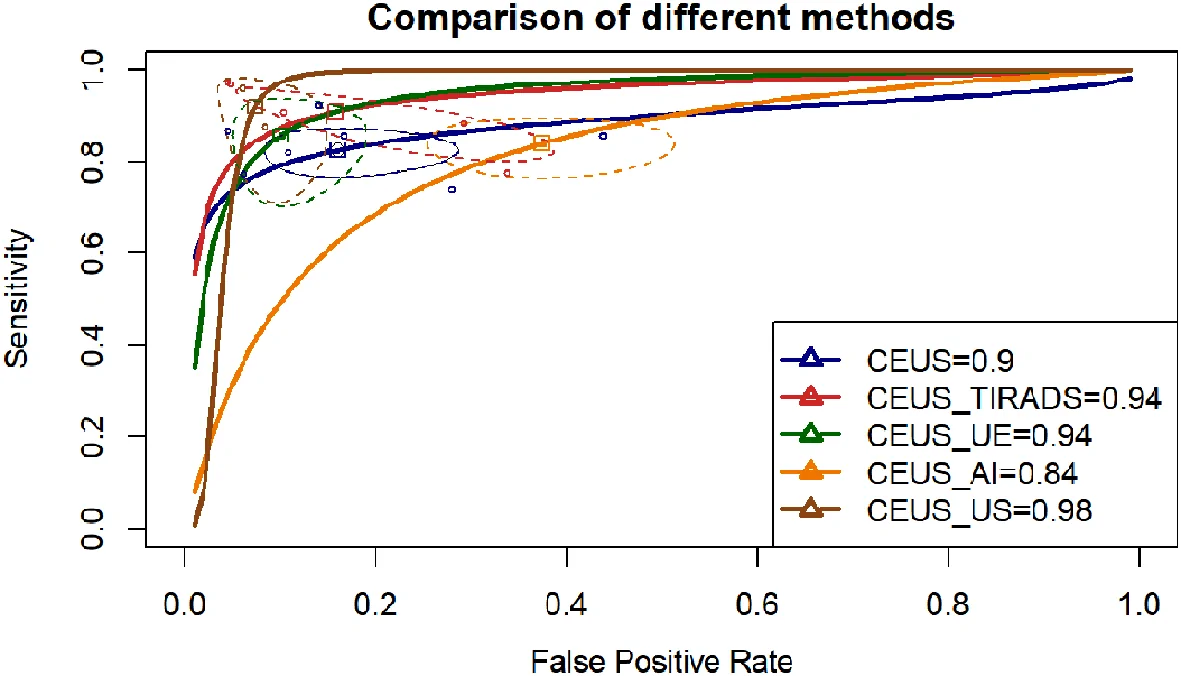
AUC values of CEUS and CEUS combined strategies in identifying non-risk-stratified TNs.

TNs underwent no risk stratification in eight studies. Specifically, the pooled SE and SP were 0.89 (95%CI 0.74-0.97) and 0.88 (95%CI 0.69-0.97) for CEUS-US, 0.90 (95%CI 0.84-0.95) and 0.80 (95%CI 0.65-0.90) for CEUS-TIRADS, 0.82 (95%CI 0.55-0.96)and 0.83 (95%CI 0.56-0.97) for CEUS-UE, 0.81 (95%CI 0.72-0.88) and 0.79 (95%CI 0.66-0.88) for CEUS, and 0.83 (95%CI 0.58-0.95) and 0.73 (95%CI 0.38-0.92) for CEUS-AI. The AUC was 0.90 (95%CI 0.85-0.96) for CEUS, 0.94 (95%CI 0.86-1.03) for CEUS-TIRADS, and 0.94 for CEUS-UE. Point estimates for AUC were 0.84 and 0.98 (95%CI -1.083-1.03), respectively, for CEUS-AI and CEUS-US (**Figure 7**).

CEUS-US had the highest SI (5.29, 95%CI 0.33-9), followed by CEUS-TIRADS (2.60, 95%CI 0.20-7), CEUS-UE (2.33, 95%CI 0.11-9), CEUS-AI (1.04, 95%CI 0.11-7), and CEUS (0.47, 95%CI 0.11-3). Approximately, the DOR was the highest for CEUS-US (109.72, 95%CI 13.21-423.46), followed by CEUS-UE (57.32, 95%CI 3.73-259.72), CEUS-TIRADS (43.64, 95%CI 13.47-98.90), CEUS-AI (27.26, 95%CI 2.25-108.30), and CEUS (19.34, 95%CI 6.88-40.81) (**Table 4**).

**Table 4:** Performance of different methods in identifying non-risk-stratified TNs.

Diagnostic techniques	SE (95%CI)	SP (95%CI)	DOR (95%CI)	SI (95%CI)	Relative SE (95%CI)	Relative SP (95%CI)
CEUS	0.81 (0.72-0.88)	0.79 (0.66-0.88)	19.34 (6.88-40.81)	0.47 (0.11-3)	1	1
CEUS-TIRADS	0.90 (0.84-0.95)	0.80 (0.65-0.90)	43.64 (13.47-98.90)	2.60 (0.20-7)	1.11 (1.01-1.26)	1.01 (0.86-1.14)
CEUS-UE	0.82 (0.55-0.96)	0.83 (0.56-0.97)	57.32 (3.73-259.72)	2.33 (0.11-9)	1.01 (0.68-1.22)	1.06 (0.72-1.29)
CEUS-AI	0.83 (0.58-0.95)	0.73 (0.38-0.92)	27.26 (2.25-108.30)	1.04 (0.11-7)	1.02 (0.72-1.21)	0.92 (0.48-1.18)
CEUS-US	0.89 (0.74-0.97)	0.88 (0.69-0.97)	109.72 (13.21-423.46)	5.29 (0.33-9)	1.09 (0.92-1.25)	1.11 (0.90-1.30)

**Figure 8:**
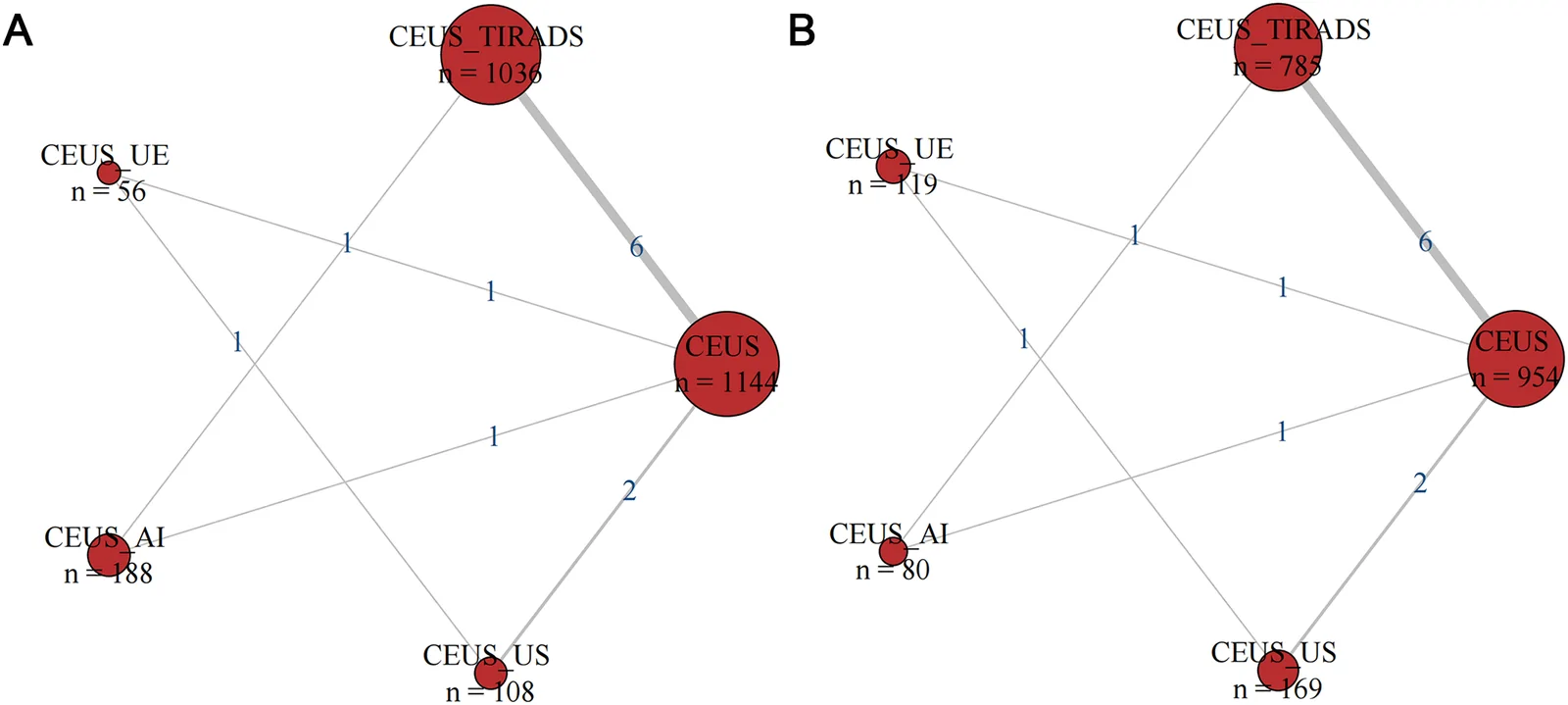
**(A)** Network diagram for SE in identifying non-risk-stratified TNs; **(B)** Network diagram for SP in identifying non-risk-stratified TNs.

For SE, CEUS-TIRADS achieved the highest SUCRA value (78%), followed by CEUS-US (61.4%), CEUS-AI (56.9%), CEUS-UE (43.4%), and CEUS (10.4%). The corresponding network diagram and league table are presented in **Figure 8A** and **Supplementary Table 7**, respectively.

For SP, CEUS-US had the highest SUCRA value (76.9%), followed by CEUS-UE (61.4%), CEUS-AI (41.4%), CEUS-TIRADS (40.2%), and CEUS (30.2%). The corresponding network diagram and league table are shown in **Figure 8B** and **Supplementary Table 8**, respectively.

In the evidence network, no closed loop was formed, precluding the consistency test using the node-splitting method. Therefore, we evaluated consistency indirectly by comparing the DIC values between the consistency and inconsistency models. The DIC differences were minimal for both SE (34.31 vs. 34.25) and SP (37.76 vs. 37.67), suggesting no substantial global inconsistency. Accordingly, we deemed the consistency assumption to be valid for this subgroup, and league tables and SUCRA values were derived subsequently based on the consistency model.

To sum up, CEUS-US demonstrated higher pooled SE, SP, SUCRA, and SI in identifying non-risk-stratified TNs. However, due to the limited number of included studies in this subgroup and the broad CI for some indicators, the heterogeneity test revealed substantial heterogeneity for SE (I²=80.6%) and low-to-moderate between-study heterogeneity for SP (I²=34.5%). These findings should be interpreted as relative trends based on current evidence. Therefore, more high-quality, direct comparative studies are warranted for further validation.

The number of included studies was limited for some diagnostic techniques (e.g., CEUS-AI, CEUS-UE), so the parameters in the bivariate model could not be identified, preventing stable estimation of the 95%CI for AUC. Therefore, only point estimates were reported. Forest plots for SE, SP, and DOR across groups are presented in **Supplementary Figures 4**-**S6**.

#### 3.5.3 Other potential sources of heterogeneity: descriptive analysis based on feature distribution

Given the uneven distribution of factors such as machine model, study type, and country across studies, as well as the limited number of included studies, these factors underwent no further subgroup analyses but were descriptively reported (**Table 5**).

**Table 5:** Original studies, number of patients and nodules stratified by machine model, study type, and country.

Type	Original study	Number of patients	Number of nodules
Country			
China	24	4,421	4,813
Romania	1	133	157
India	1	50	52
Machine			
Philips iU22	8	1,664	1,867
Siemens	7	1,347	1,535
GE LOGIQ	7	1,077	1,163
Esaote	5	850	894
SuperSonic	5	800	924
Hitachi	1	338	378
Mindray	1	209	268
Samsung	2	322	384
Toshiba Aplio 500	1	116	116
Study type			
Retrospective cohort	19	3,764	4,196
Prospective cohort	7	720	826

## 4 Discussion

This study ultimately included five imaging diagnostic techniques: CEUS alone, CEUS-TIRADS, CEUS-UE, CEUS-AI, and CEUS-US. Based on the comprehensive assessment of relative SE, relative SP, SI, DOR, AUC, and SUCRA rankings, CEUS-UE showed the most favorable overall diagnostic performance, and CEUS-AI had promising diagnostic performance for identifying high-risk TNs with suspicious features. However, given the limited number of studies, this finding should be interpreted cautiously and requires further external validation. Similarly, CEUS-US demonstrated favorable diagnostic performance in identifying non-risk-stratified TNs. This result requires confirmation in larger cohorts. Overall, the optimal diagnostic strategy may vary according to different risk levels. Therefore, more individualized management of TNs is needed. Additionally, the robustness of the comparative rankings was further evaluated using SUCRA. Although there were some minor differences between the SUCRA rankings and the relative SE- and SP-based rankings, the overall trends were largely similar. Since SUCRA was estimated separately for SE and SP, slight differences from the corresponding rankings based on relative efficacy were not unexpected. The SI combines SE and SP into a single measure, whereas AUC reflects the overall discriminatory power of a diagnostic technique for benign and malignant TNs. These differences may partly account for the variations in ranking. Besides, we also considered using the league table to facilitate a more comprehensive comparison between techniques. Collectively, the SI, SUCRA, AUC, and league table results showed generally similar trends and provided complementary information for the comparison of diagnostic performance of different techniques.

The meta-analysis of the 26 studies revealed consistently high AUC values of CEUS, suggesting its value in the differential diagnosis of benign and malignant TNs. Consistent with our findings, a meta-analysis (39) showed that CEUS was highly accurate in diagnosing benign and malignant TNs, with a pooled SE of 0.86 (95%CI 0.83-0.89), a pooled SP of 0.86 (95%CI 0.82-0.89), and an overall AUC of 0.93. Yu et al. (40) found that CEUS can assess the status of cervical lymph nodes and determine whether cervical lymph node metastasis (CLNM) occurs in thyroid carcinoma. The variation in perfusion patterns had the highest accuracy in identifying CLNM, with pooled SE, SP, and AUC of 0.95 (0.91, 0.97), 0.87 (0.69, 0.96), and 0.96 (0.94, 0.98), respectively. Additionally, Zhang et al. (41) discussed heterogeneity in meta-analyses of CEUS. They argued that great heterogeneity arose from the nodule size and CEUS mode (qualitative diagnosis, quantitative or semi-quantitative parameters). They also highlighted the higher diagnostic value of quantitative parameters than qualitative diagnosis (42). However, they did not deny the importance of CEUS in distinguishing benign and malignant TNs. Interestingly, this study also revealed that CEUS alone had good diagnostic efficacy in distinguishing benign and malignant TNs, suggesting that it may still hold significant and independent clinical value. Our findings may offer new references for the current research direction for the imaging diagnosis of TNs, i.e., the diagnostic potential of CEUS alone should also be re-assessed while emphasizing multimodal imaging. Of course, given the heterogeneity across studies and the need to further clarify the specific target population and optimal use scenarios, more high-quality studies are still required in the future to validate these findings.

UE detects tissue stiffness by internal or external deformation stimulation and shear wave imaging. It is classified into early SRE and SWE based on the emergence of the acoustic radiation force impulse technique (43, 44). SWE significantly improves accuracy by eliminating the manual force applied to the transducer. UE can assess nodule hardness, while CEUS helps assess vascular perfusion and hemodynamics of nodules (5). UE distinguishes benign and malignant lesions primarily by quantitative parameters such as Young’s modulus (E) (26), SWV (33), and Rago’s five-point scale (31). Following the parallel rule, CEUS-UE determines malignant nodules if either method indicates malignancy. This rule increases diagnostic SE to 0.92 (95%CI 0.88-0.95), and also produces satisfactory pooled SP (0.83, 95%CI 0.75-0.89) and AUC (0.97, 95%CI 0.99-0.96). Presumably, CEUS-UE can detect early-stage malignant lesions with low blood supply but high stiffness, or malignant nodules with non-extreme stiffness but abnormal perfusion due to cystic lesions. A meta-analysis (45) published in *Nature* also demonstrated that CEUS-UE compensates for the deficiencies of a single technique, which can offer more diagnostic information from perspectives of blood perfusion and tissue stiffness, with a pooled SE of 0.93, a pooled SP of 0.91, and an AUC of 0.97. A multicenter study identified four independent risk factors for CEUS and UE: irregular shapes, ESS (≥3 points), absence of circular enhancement, and blurred boundaries post-enhancement. It also created a predictive model, yielding AUCs of 0.936 and 0.902, respectively, in training and validation sets, demonstrating strong clinical utility (46).

CEUS-AI was effective in identifying high-risk TNs. Based on US, this study utilized the AI-SONIC™ system (16, 22), ITS100 system (11) or S-Detect software (19) as AI techniques to assess morphological characteristics of nodules (e.g., boundary, shape, and echo), ultimately obtaining the probability of benign/malignant TNs. Then, combined with malignant characteristics shown by CEUS, assignment summation (16, 19) or majority voting system (11) was used to determine the probability of benign/malignant TNs. During system or software evaluation, TIRADS stratification (19) may be referenced. High-risk nodules often exhibit complex imaging features, irregular boundaries, and internal heterogeneity. Consequently, manual reading is prone to experience-dependent or threshold bias. In contrast, AI can mitigate subjective differences in manual reading, greatly helping less experienced physicians (47). A meta-analysis by Zhan et al. (48) revealed that higher malignancy rates of TNs correspond to stronger performance of AI models. Specifically, when the malignancy rate of TNs was ≥50%, the AI model had an SE of 0.90 (0.88-0.92) and an SP of 0.88 (0.84-0.92), highly similar to our findings. To sum up, CEUS-AI exhibits a greater advantage in populations with a high probability of malignancy. Despite the excellent performance of computer-aided diagnostic systems, the greater contribution of experienced operators remains undeniable (49).

In this study, TIRADS was based on US images in cohort studies, but CEUS-US relied solely on raw US images without reference to TIRADS. The malignancy of TNs was assessed by parallel interpretation of structure (US) and function (CEUS) or multivariate logistic regression. Therefore, CEUS-US was grouped separately. Original structures of US (e.g., hypoecho, aspect ratio ≥1, blurred boundaries, microcalcification (7, 17, 18, 36), high-grade blood flow (17), suspicious cervical lymph nodes (36)) can be combined with qualitative signs or quantitative parameters like TTP (7) of CEUS to yield higher SI in non-risk-stratified populations. This finding likely stems from the complementary information of morphology (US) and microvascular perfusion (CEUS), enhancing the discriminatory power of the combined strategy across a broader patient spectrum (50). Notably, the non-risk-stratified population exhibited high heterogeneity. Thus, the relative advantage of CEUS-US in this subgroup should be interpreted as a trend rather than an unconditional, universally applicable advantage.

This study included five CEUS-based diagnostic techniques and their associated data for the first time to investigate their value in distinguishing benign and malignant TNs. CEUS-TIRADS and CEUS-US were also methodologically distinguished, avoiding confusion between “combined scoring based on a stratification system” and “combination based on raw structural-functional information fusion”. This enhances the interpretability of node definitions. Additionally, subgroup analyses were conducted by the risk level. The results suggest that we should interpret enhancement of diagnostic accuracy in different clinical settings, and for TNs, we should maximize diagnostic sensitivity, also take into account specificity, and avoid overdiagnosis, thereby reducing unnecessary interventional procedures.

Some limitations are worth noting. First, methodological heterogeneity was significant across the original studies, including inconsistencies in CEUS judgement criteria, qualitative and quantitative assessment methods, types and thresholds of elastography techniques, AI systems and thresholds, and combination rules. Second, several subgroup analyses were conducted based on a limited number of studies, particularly CEUS-AI in the high-risk subgroup and CEUS-US in the non-risk-stratified subgroup. Consequently, the corresponding CIs were relatively broad, indicating considerable statistical uncertainty in these estimates. Therefore, our findings should be interpreted cautiously and regarded as suggestive rather than definitive, and should be validated in larger prospective studies. Third, the regional heterogeneity of the included studies may limit the external validity of our findings. Of the 26 included studies, 24 were conducted in China, while the remaining two were from Romania and India, respectively. Given that the diagnostic performance of CEUS is highly operator-dependent and that the prevalence of papillary thyroid carcinoma as well as the characteristics of TNs may differ among Western populations, the generalizability of our findings may be limited. Therefore, further validation in North American, European, and other non-Asian populations is warranted. Finally, the applicability of CEUS combined strategies may be limited in sites lacking CEUS equipment or with limited experience of operators, thus affecting their reproducibility across different settings. Therefore, more prospective, multicenter, international studies with more standardized methodologies are required in the future.

## 5 Conclusion

In conclusion, CEUS-UE possesses the strongest overall diagnostic efficacy. CEUS, a functional technique providing information of microcirculatory perfusion, exhibits complementary value to ultrasound structural signs, elastic hardness assessment, and intelligent morphological identification. Therefore, combined with TIRADS classification and decision-making objectives, individualized diagnostic strategies can be selected in clinical practice. Given the limitations of this study, more prospective, multicenter studies with standardized parameters and unified combined strategies are required in the future to further validate and enhance the generalizability and clinical benefits of CEUS combined strategies.

## Data Availability

The original contributions presented in the study are included in the article/**Supplementary Material**. Further inquiries can be directed to the corresponding author.
