# Supplementary material for: Diagnostic value of contrast-enhanced ultrasound combined with other imaging techniques in differentiating benign and malignant thyroid nodules: a network meta-analysis
Source: Front Endocrinol (Lausanne). 2026 Sep 9;17:1887134. doi: 10.3389/fendo.2026.1887134 (PMC13597244; doi:10.3389/fendo.2026.1887134)
Supplement: Supplementary file 1 [file SupplementaryFile1.docx]

**Supplementary Material**

**Table S1** Electronic search strategies

**Table S2** Potential scale reduction factor (PSRF) values for convergence assessment.

**Table S3** League table of sensitivity for TNs

**Table S4** League table of specificity for TNs

**Table S5** League table of sensitivity for high-risk TNs

**Table S6** League table of specificity for high-risk TNs

**Table S7** League table of sensitivity for non-risk-stratified TNs

**Table S8** League table of specificity for non-risk-stratified TNs

**Figure S1** R-hat values in NMA

**Figure S2** Node-splitting plot in TNs

**Figure S3** Node-splitting plot in high-risk TNs

**Figure S4** Forest plot for sensitivity in NMA

**Figure S5** Forest plot for specificity in NMA

**Figure S6** Forest plot for DOR in NMA

**Table S1. Electronic search strategies**

| **PubMed: 257records** | |
| --- | --- |
| **#1** | (ThyroidNodule[MeSHTerms])OR("colloidnodule(thyroid)"[Title/Abstract]OR"colloidalnodule(thyroid)"[Title/Abstract]OR"noduleofthethyroidgland"[Title/Abstract]OR"noduleofthyroidgland"[Title/Abstract]OR"thyroidcolloidnodule"[Title/Abstract]OR"thyroidcolloidalnodule"[Title/Abstract]OR"thyroidglandnodule"[Title/Abstract]OR"thyroidnodule"[Title/Abstract]OR"ThyroidNodules"[Title/Abstract]OR"thyroidsolitarynodule"[Title/Abstract]OR"thyroidalglandnodule"[Title/Abstract]OR"thyroidalnodule"[Title/Abstract]OR"thyroidealnodule"[Title/Abstract]OR"ThyroidNeoplasms"[Title/Abstract]) |
| **#2** | "contrast-enhancedultrasound"[Title/Abstract]OR"CEUS(echography)"[Title/Abstract]OR"contrastenhancedultrasound"[Title/Abstract]OR"contrast-enhancedultrasonography"[Title/Abstract] |
| **#3** | "ComputerEchotomography"[Title/Abstract]OR"diagnosticultrasonicexamination"[Title/Abstract]OR"diagnosticultrasonicimaging"[Title/Abstract]OR"diagnosticultrasonicmethod"[Title/Abstract]OR"DiagnosticUltrasound"[Title/Abstract]OR"DiagnosticUltrasounds"[Title/Abstract]OR"doptone"[Title/Abstract]OR"duplexechography"[Title/Abstract]OR"echogram"[Title/Abstract]OR"echographicevaluation"[Title/Abstract]OR"Echography"[Title/Abstract]OR"echoscopy"[Title/Abstract]OR"echosound"[Title/Abstract]OR"Echotomography"[Title/Abstract]OR"highresolutionechography"[Title/Abstract]OR"MedicalSonography"[Title/Abstract]OR"sonogram"[Title/Abstract]OR"sonographicexamination"[Title/Abstract]OR"sonographicscreening"[Title/Abstract]OR"sonography"[Title/Abstract]OR"ultrasonicdetection"[Title/Abstract]OR"UltrasonicDiagnoses"[Title/Abstract]OR"ultrasonicdiagnosis"[Title/Abstract]OR"ultrasonicecho"[Title/Abstract]OR"ultrasonicexamination"[Title/Abstract]OR"UltrasonicImaging"[Title/Abstract]OR"ultrasonicscanning"[Title/Abstract]OR"ultrasonicscintillation"[Title/Abstract]OR"UltrasonicTomography"[Title/Abstract]OR"ultrasonogram"[Title/Abstract]OR"ultrasonographicexamination"[Title/Abstract]OR"UltrasonographicImaging"[Title/Abstract]OR"UltrasonographicImagings"[Title/Abstract]OR"ultrasonographicscreening"[Title/Abstract]OR"Ultrasonography"[Title/Abstract]OR"ultrasounddiagnosis"[Title/Abstract]OR"UltrasoundImaging"[Title/Abstract]OR"ultrasoundscanning"[Title/Abstract] |
| **#4** | "contrast-enhanced"[Title/Abstract]OR"contrastenhanced"[Title/Abstract] |
| **#5** | #2OR(#3AND#4) |
| **#6** | #1AND#5 |
| **Embase 490records** | |
| **1#** | 'thyroid nodule'/exp |
| **2#** | 'colloid nodule (thyroid)':ab,ti,kw OR 'colloidal nodule (thyroid)':ab,ti,kw OR 'nodule of the thyroid gland':ab,ti,kw OR 'nodule of thyroid gland':ab,ti,kw OR 'thyroid colloid nodule':ab,ti,kw OR 'thyroid colloidal nodule':ab,ti,kw OR 'thyroid gland nodule':ab,ti,kw OR 'thyroid nodule':ab,ti,kw OR 'thyroid nodules':ab,ti,kw OR 'thyroid solitary nodule':ab,ti,kw OR 'thyroidal gland nodule':ab,ti,kw OR 'thyroidal nodule':ab,ti,kw OR 'thyroideal nodule':ab,ti,kw OR 'thyroid neoplasms':ab,ti,kw |
| **3#** | "ComputerEchotomography"[Title/Abstract]OR"diagnosticultrasonicexamination"[Title/Abstract]OR"diagnosticultrasonicimaging"[Title/Abstract]OR"diagnosticultrasonicmethod"[Title/Abstract]OR"DiagnosticUltrasound"[Title/Abstract]OR"DiagnosticUltrasounds"[Title/Abstract]OR"doptone"[Title/Abstract]OR"duplexechography"[Title/Abstract]OR"echogram"[Title/Abstract]OR"echographicevaluation"[Title/Abstract]OR"Echography"[Title/Abstract]OR"echoscopy"[Title/Abstract]OR"echosound"[Title/Abstract]OR"Echotomography"[Title/Abstract]OR"highresolutionechography"[Title/Abstract]OR"MedicalSonography"[Title/Abstract]OR"sonogram"[Title/Abstract]OR"sonographicexamination"[Title/Abstract]OR"sonographicscreening"[Title/Abstract]OR"sonography"[Title/Abstract]OR"ultrasonicdetection"[Title/Abstract]OR"UltrasonicDiagnoses"[Title/Abstract]OR"ultrasonicdiagnosis"[Title/Abstract]OR"ultrasonicecho"[Title/Abstract]OR"ultrasonicexamination"[Title/Abstract]OR"UltrasonicImaging"[Title/Abstract]OR"ultrasonicscanning"[Title/Abstract]OR"ultrasonicscintillation"[Title/Abstract]OR"UltrasonicTomography"[Title/Abstract]OR"ultrasonogram"[Title/Abstract]OR"ultrasonographicexamination"[Title/Abstract]OR"UltrasonographicImaging"[Title/Abstract]OR"UltrasonographicImagings"[Title/Abstract]OR"ultrasonographicscreening"[Title/Abstract]OR"Ultrasonography"[Title/Abstract]OR"ultrasounddiagnosis"[Title/Abstract]OR"UltrasoundImaging"[Title/Abstract]OR"ultrasoundscanning"[Title/Abstract] |
| **4#** | "contrast-enhanced"[Title/Abstract]OR"contrastenhanced"[Title/Abstract] |
| **5#** | #2OR(#3AND#4) |
| **6#** | #1AND#5 |
| **Embase 490records** | |
| **1#** | 'thyroid nodule'/exp |
| **2#** | 'colloid nodule (thyroid)':ab,ti,kw OR 'colloidal nodule (thyroid)':ab,ti,kw OR 'nodule of the thyroid gland':ab,ti,kw OR 'nodule of thyroid gland':ab,ti,kw OR 'thyroid colloid nodule':ab,ti,kw OR 'thyroid colloidal nodule':ab,ti,kw OR 'thyroid gland nodule':ab,ti,kw OR 'thyroid nodule':ab,ti,kw OR 'thyroid nodules':ab,ti,kw OR 'thyroid solitary nodule':ab,ti,kw OR 'thyroidal gland nodule':ab,ti,kw OR 'thyroidal nodule':ab,ti,kw OR 'thyroideal nodule':ab,ti,kw OR 'thyroid neoplasms':ab,ti,kw |
| **3#** | 'contrast-enhanced ultrasound'/exp |
| **4#** | 'contrast-enhanced ultrasound':ab,ti,kw OR 'ceus (echography)':ab,ti,kw OR 'contrast enhanced ultrasound':ab,ti,kw OR 'contrast-enhanced ultrasonography':ab,ti,kw |
| **5#** | 'computer echotomography':ab,ti,kw OR 'diagnostic ultrasonic examination':ab,ti,kw OR 'diagnostic ultrasonic imaging':ab,ti,kw OR 'diagnostic ultrasonic method':ab,ti,kw OR 'diagnostic ultrasound':ab,ti,kw OR 'diagnostic ultrasounds':ab,ti,kw OR 'doptone':ab,ti,kw OR 'duplex echography':ab,ti,kw OR 'echogram':ab,ti,kw OR 'echographic evaluation':ab,ti,kw OR 'echography':ab,ti,kw OR 'echoscopy':ab,ti,kw OR 'echosound':ab,ti,kw OR 'echotomography':ab,ti,kw OR 'high resolution echography':ab,ti,kw OR 'medical sonography':ab,ti,kw OR 'sonogram':ab,ti,kw OR 'sonographic examination':ab,ti,kw OR 'sonographic screening':ab,ti,kw OR 'sonography':ab,ti,kw OR 'ultrasonic detection':ab,ti,kw OR 'ultrasonic diagnoses':ab,ti,kw OR 'ultrasonic diagnosis':ab,ti,kw OR 'ultrasonic echo':ab,ti,kw OR 'ultrasonic examination':ab,ti,kw OR 'ultrasonic imaging':ab,ti,kw OR 'ultrasonic scanning':ab,ti,kw OR 'ultrasonic scintillation':ab,ti,kw OR 'ultrasonic tomography':ab,ti,kw OR 'ultrasonogram':ab,ti,kw OR 'ultrasonographic examination':ab,ti,kw OR 'ultrasonographic imaging':ab,ti,kw OR 'ultrasonographic imagings':ab,ti,kw OR 'ultrasonographic screening':ab,ti,kw OR 'ultrasonography':ab,ti,kw OR 'ultrasound diagnosis':ab,ti,kw OR 'ultrasound imaging':ab,ti,kw OR 'ultrasound scanning':ab,ti,kw |
| **6#** | 'contrast-enhanced':ab,ti,kw OR 'contrast enhanced':ab,ti,kw |
| **7#** | #5 AND #6 |
| **8#** | #4 OR #7 |
| **9#** | #3 OR #8 |
| **10#** | #1 OR #2 |
| **11#** | #9 AND #10 |
| **Web of Science 514records** | |
| **1#** | (TS=("colloid nodule (thyroid)" OR "colloidal nodule (thyroid)" OR nodule of the thyroid gland OR nodule of thyroid gland OR thyroid colloid nodule OR thyroid colloidal nodule OR thyroid gland nodule OR thyroid nodule OR Thyroid Nodules OR thyroid solitary nodule OR thyroidal gland nodule OR thyroidal nodule OR thyroideal nodule OR Thyroid Neoplasms)) AND TS=((“colloid nodule (thyroid)” OR “colloidal nodule (thyroid)” OR nodule of the thyroid gland OR nodule of thyroid gland OR thyroid colloid nodule OR thyroid colloidal nodule OR thyroid gland nodule OR thyroid nodule OR Thyroid Nodules OR thyroid solitary nodule OR thyroidal gland nodule OR thyroidal nodule OR thyroideal nodule OR Thyroid Neoplasms)) |
| **2#** | (TS=(contrast-enhanced ultrasound OR “CEUS (echography)” OR contrast enhanced ultrasound OR contrast-enhanced ultrasonography)) |
| **3#** | (TS=(contrast-enhanced OR contrast enhanced)) |
| **4#** | (TS=(Computer Echotomography OR diagnostic ultrasonic examination OR diagnostic ultrasonic imaging OR diagnostic ultrasonic method OR Diagnostic Ultrasound OR Diagnostic Ultrasounds OR doptone OR duplex echography OR echogram OR echographic evaluation OR Echography OR echoscopy OR echosound OR Echotomography OR high resolution echography OR Medical Sonography OR sonogram OR sonographic examination OR sonographic screening OR sonography OR ultrasonic detection OR Ultrasonic Diagnoses OR ultrasonic diagnosis OR ultrasonic echo OR ultrasonic examination OR Ultrasonic Imaging OR ultrasonic scanning OR ultrasonic scintillation OR Ultrasonic Tomography OR ultrasonogram OR ultrasonographic examination OR Ultrasonographic Imaging OR Ultrasonographic Imagings OR ultrasonographic screening OR Ultrasonography OR ultrasound diagnosis OR Ultrasound Imaging OR ultrasound scanning)) |
| **5#** | #3 AND #4 |
| **6#** | #5 OR #2 |
| **7#** | #6 AND #1 |

**Table S2 Potential scale reduction factor (PSRF) values for convergence assessment.**

| Analysis | PRSF值 |
| --- | --- |
| sensitivity in TNs | 1 |
| specificity in TNs | 1 |
| sensitivity in high-risk TNs | 1 |
| specificity in high-risk TNs. | 1 |
| sensitivity in non-risk-stratified TNs. | 1 |
| specificity in non-risk-stratified TNs. | 1 |

**Table S3 League table of sensitivity for TNs**

| **Diagnostic techniques** | **CEUS**  **OR(95CrI)** | **CEUS_TIRADS**  **OR(95CrI)** | **CEUS_UE**  **OR(95CrI)** | **CEUS_AI**  **OR(95CrI)** | **CEUS_US**  **OR(95CrI)** |
| --- | --- | --- | --- | --- | --- |
| CEUS | 1 | 2.49 (1.5, 4.34) | 3.27 (1.73, 6.3) | 3.3 (1.46, 8.12) | 1.25 (0.54, 2.95) |
| CEUS_TIRADS | 0.4 (0.23, 0.67) | 2 | 1.31 (0.56, 2.96) | 1.33 (0.53, 3.36) | 0.5 (0.18, 1.34) |
| CEUS_UE | 0.31 (0.16, 0.58) | 0.76 (0.34, 1.78) | 3 | 1.01 (0.36, 3.05) | 0.38 (0.14, 1.05) |
| CEUS_AI | 0.3 (0.12, 0.69) | 0.75 (0.3, 1.87) | 0.99 (0.33, 2.8) | 4 | 0.38 (0.11, 1.24) |
| CEUS_US | 0.8 (0.34, 1.84) | 2 (0.74, 5.48) | 2.63 (0.95, 7.15) | 2.65 (0.81, 9.05) | 5 |

**Table S4 League table of specificity for TNs**

| **Diagnostic techniques** | **CEUS**  **OR(95CrI)** | **CEUS_TIRADS**  **OR(95CrI)** | **CEUS_UE**  **OR(95CrI)** | **CEUS_AI**  **OR(95CrI)** | **CEUS_US**  **OR(95CrI)** |
| --- | --- | --- | --- | --- | --- |
| CEUS | 1 | 0.86 (0.7, 1.06) | 1.09 (0.79, 1.5) | 0.87 (0.65, 1.17) | 2.36 (1.51, 3.77) |
| CEUS_TIRADS | 1.16 (0.95, 1.42) | 2 | 1.26 (0.87, 1.84) | 1.01 (0.75, 1.38) | 2.73 (1.68, 4.57) |
| CEUS_UE | 0.92 (0.67, 1.26) | 0.79 (0.54, 1.15) | 3 | 0.8 (0.52, 1.24) | 2.17 (1.28, 3.72) |
| CEUS_AI | 1.15 (0.85, 1.53) | 0.99 (0.73, 1.34) | 1.25 (0.81, 1.93) | 4 | 2.7 (1.58, 4.71) |
| CEUS_US | 0.42 (0.27, 0.66) | 0.37 (0.22, 0.6) | 0.46 (0.27, 0.78) | 0.37 (0.21, 0.63) | 5 |

**Table S5 League table of sensitivity for high-risk TNs**

| **Diagnostic techniques** | **CEUS**  **OR(95CrI)** | **CEUS_TIRADS**  **OR(95CrI)** | **CEUS_UE**  **OR(95CrI)** | **CEUS_AI**  **OR(95CrI)** | **CEUS_US**  **OR(95CrI)** |
| --- | --- | --- | --- | --- | --- |
| CEUS | 1 | 2.32 (1.53, 3.6) | 3.81 (2.6, 5.68) | 4.4 (2.33, 8.85) | 0.72 (0.37, 1.38) |
| CEUS_TIRADS | 0.43 (0.28, 0.65) | 2 | 1.64 (0.92, 2.93) | 1.9 (0.91, 4.15) | 0.31 (0.14, 0.67) |
| CEUS_UE | 0.26 (0.18, 0.38) | 0.61 (0.34, 1.09) | 3 | 1.16 (0.54, 2.54) | 0.19 (0.09, 0.4) |
| CEUS_AI | 0.23 (0.11, 0.43) | 0.53 (0.24, 1.1) | 0.87 (0.39, 1.84) | 4 | 0.16 (0.06, 0.41) |
| CEUS_US | 1.39 (0.72, 2.7) | 3.23 (1.49, 7.12) | 5.31 (2.5, 11.43) | 6.14 (2.45, 16.09) | 5 |

**Table S6 League table of specificity for high-risk TNs**

| **Diagnostic techniques** | **CEUS**  **OR(95CrI)** | **CEUS_TIRADS**  **OR(95CrI)** | **CEUS_UE**  **OR(95CrI)** | **CEUS_AI**  **OR(95CrI)** | **CEUS_US**  **OR(95CrI)** |
| --- | --- | --- | --- | --- | --- |
| CEUS | 1 | 0.88 (0.42, 1.97) | 0.93 (0.44, 1.95) | 1.11 (0.43, 3.27) | 3.17 (0.98, 10.62) |
| CEUS_TIRADS | 1.14 (0.51, 2.37) | 2 | 1.06 (0.35, 2.99) | 1.28 (0.41, 4.13) | 3.63 (0.87, 14.44) |
| CEUS_UE | 1.07 (0.51, 2.25) | 0.94 (0.33, 2.83) | 3 | 1.2 (0.36, 4.44) | 3.4 (0.85, 14.08) |
| CEUS_AI | 0.9 (0.31, 2.3) | 0.78 (0.24, 2.42) | 0.83 (0.23, 2.76) | 4 | 2.83 (0.57, 12.97) |
| CEUS_US | 0.32 (0.09, 1.02) | 0.28 (0.07, 1.15) | 0.29 (0.07, 1.17) | 0.35 (0.08, 1.76) | 5 |

**Table S7 League table of sensitivity for non-risk-stratified TNs**

| **Diagnostic techniques** | **CEUS**  **OR(95CrI)** | **CEUS_TIRADS**  **OR(95CrI)** | **CEUS_UE**  **OR(95CrI)** | **CEUS_AI**  **OR(95CrI)** | **CEUS_US**  **OR(95CrI)** |
| --- | --- | --- | --- | --- | --- |
| CEUS | 1 | 1.93 (1.52, 2.48) | 1.37 (0.52, 3.83) | 1.6 (0.99, 2.64) | 1.69 (0.7, 4.33) |
| CEUS_TIRADS | 0.52 (0.4, 0.66) | 2 | 0.71 (0.26, 2.03) | 0.83 (0.51, 1.37) | 0.87 (0.35, 2.31) |
| CEUS_UE | 0.73 (0.26, 1.93) | 1.41 (0.49, 3.85) | 3 | 1.17 (0.38, 3.49) | 1.23 (0.41, 3.62) |
| CEUS_AI | 0.62 (0.38, 1.01) | 1.21 (0.73, 1.97) | 0.86 (0.29, 2.66) | 4 | 1.05 (0.38, 3.01) |
| CEUS_US | 0.59 (0.23, 1.44) | 1.15 (0.43, 2.86) | 0.81 (0.28, 2.43) | 0.95 (0.33, 2.62) | 5 |

**Table S8 League table of specificity for non-risk-stratified TNs**

| **Diagnostic techniques** | **CEUS**  **OR(95CrI)** | **CEUS_TIRADS**  **OR(95CrI)** | **CEUS_UE**  **OR(95CrI)** | **CEUS_AI**  **OR(95CrI)** | **CEUS_US**  **OR(95CrI)** |
| --- | --- | --- | --- | --- | --- |
| CEUS | 1 | 1.11 (0.37, 2.6) | 1.67 (0.2, 15.41) | 1.12 (0.14, 8.03) | 2.19 (0.41, 12.93) |
| CEUS_TIRADS | 0.9 (0.38, 2.68) | 2 | 1.5 (0.17, 18.76) | 1.01 (0.14, 8.03) | 1.99 (0.32, 16.73) |
| CEUS_UE | 0.6 (0.06, 4.92) | 0.67 (0.05, 6.05) | 3 | 0.67 (0.03, 11.7) | 1.31 (0.15, 11.77) |
| CEUS_AI | 0.89 (0.12, 7) | 0.99 (0.12, 7) | 1.5 (0.09, 32.17) | 4 | 1.97 (0.15, 30.93) |
| CEUS_US | 0.46 (0.08, 2.41) | 0.5 (0.06, 3.09) | 0.76 (0.08, 6.82) | 0.51 (0.03, 6.56) | 5 |

**Figure S1 R-hat values in NMA**

Figure S1 (A) Patients with TNs

Figure S1 (B) Patients with high-risk TNs

Figure S1 (C) Patients with non-risk-stratified TNs

**Figure S2** Node-splitting plot in TNs

**Figure S2(A) Node-splitting plot for sensitivity in TNs**

**Figure S2(B) Node-splitting plot for specificity in TNs**

**Figure S3** Node-splitting plot in high-risk TNs

**Figure S3(A) Node-splitting plot for sensitivity in high-risk TNs**

**Figure S3(B) Node-splitting plot for specificity in high-risk TNs**

**Figure S4 Forest plot for sensitivity in NMA**

Figure S4 (A) Patients with TNs

Figure S4 (B) Patients with high-risk TNs

Figure S4 (C) Patients with non-risk-stratified TNs

**Figure S5 Forest plot for specificity in NMA**

Figure S5 (A) Patients with TNs

Figure S5 (B) Patients with high-risk TNs

Figure S5 (C) Patients with non-risk-stratified TNs

**Figure S5 Forest plot for DOR in NMA**

Figure S6 (A) Patients with TNs

Figure S6 (B) Patients with high-risk TNs

Figure S6 (C) Patients with non-risk-stratified TNs
